# Changes in depression and suicide thoughts among doctors and nurses in Europe: a repeated cross-sectional study

**DOI:** 10.1192/j.eurpsy.2026.10363

**Published:** 2026-07-31

**Authors:** V. A. Arrona Gómez, J. L. Ayuso Mateos

**Affiliations:** 1 Department of Psychiatry, Universidad Autónoma de Madrid; 2 Centro de Investigación Biomédica en Red en Salud Mental, Madrid, Spain

## Abstract

**Introduction:**

Since 2020, hundreds of studies have analysed the mental health of doctors and nurses worldwide. Initial causal claims of pandemic-related effects were hindered by the scarcity of comparable pre-pandemic studies. To address this gap and ensure continued surveillance, the World Health Organization Regional Office for Europe launched a survey for all doctors and nurses practising in the EU, Iceland and Norway (MeND).

**Objectives:**

We compare the prevalence of depressive symptoms and suicidal thoughts from 2020 through 2025, in a sample of doctors and nurses from Czechia, Italy, the Netherlands, and Spain.

**Methods:**

We conducted a repeated cross-sectional study. Data was obtained from the HEROES (2020-2021) and MeND (2024-2025) cohorts. We used self-reported measures of depressive symptoms (i.e, PHQ-9) and suicidal thoughts, and key occupational exposures (working hours, support from colleagues, managing anger from patients and relatives, and exposure to workplace violence). We compared the proportion of individuals across exposures and outcomes between the two periods through multivariate regression analyses.

**Results:**

We included 39,533 respondents (5,184 from HEROES and 34,349 from MeND). Most participants were from Italy, followed by Spain, Czechia and, finally, the Netherlands. Compared to 2020-2021, individuals reported fewer weekly working hours, greater exposure to angry patients and/or relatives, lower exposure to general violence, and similar levels of colleague support in 2024-2025. The prevalence of depressive symptoms and suicidal ideation was higher in 2024-2025 compared to 2020-2021.

**Conclusions:**

Doctors and nurses in Europe report higher levels of depressive symptoms and suicidal thoughts than they did in 2020-2021. Despite the limitations of repeated cross-sectional designs, these findings highlight the need for continued monitoring of healthcare workers’ mental health and for scalable support interventions at individual, interpersonal, and organisation levels to inform policy and practice.

**Disclosure of Interest:**

None Declared

